# USP17 is required for clathrin mediated endocytosis of epidermal growth factor receptor

**DOI:** 10.18632/oncotarget.2165

**Published:** 2014-07-03

**Authors:** Jakub Jaworski, Michelle de la Vega, Sarah J. Fletcher, Cheryl McFarlane, Michelle K. Greene, Andrew W. Smyth, Sandra Van Schaeybroeck, James A. Johnston, Christopher J. Scott, Joshua Z. Rappoport, James F. Burrows

**Affiliations:** ^1^ School of Pharmacy, Queen's University Belfast, Belfast, UK; ^2^ Centre for Infection and Immunity, School of Medicine, Dentistry and Biomedical Sciences, Queen's University Belfast, Health Sciences Building, Belfast, UK; ^3^ School of Biosciences, University of Birmingham, Edgbaston, Birmingham, UK; ^4^ Centre for Cancer Research and Cell Biology, School of Medicine, Dentistry and Biomedical Sciences, Queen's University Belfast, Belfast, UK; ^5^ Current address, Inflammation Research, Amgen Inc., Thousand Oaks, CA

**Keywords:** Clathrin, Deubiquitinating, Endocytosis, Epidermal growth factor receptor, USP17

## Abstract

Previously we have shown that expression of the deubiquitinating enzyme USP17 is required for cell proliferation and motility. More recently we reported that USP17 deubiquitinates RCE1 isoform 2 and thus regulates the processing of ‘CaaX’ motif proteins. Here we now show that USP17 expression is induced by epidermal growth factor and that USP17 expression is required for clathrin mediated endocytosis of epidermal growth factor receptor. In addition, we show that USP17 is required for the endocytosis of transferrin, an archetypal substrate for clathrin mediated endocytosis, and that USP17 depletion impedes plasma membrane recruitment of the machinery required for clathrin mediated endocytosis. Thus, our data reveal that USP17 is necessary for epidermal growth factor receptor and transferrin endocytosis via clathrin coated pits, indicate this is mediated via the regulation of the recruitment of the components of the endocytosis machinery and suggest USP17 may play a general role in receptor endocytosis.

## INTRODUCTION

Ubiquitin is now recognised as an essential post-translational modification that can have diverse effects on the proteins to which it is conjugated, including tagging them for proteasomal or lysosomal degradation, facilitating protein-protein interactions, or regulating their localisation within the cell [1]. Six families of deubiquitinating enzymes consisting of at least 98 members have now been identified and implicated in the regulation of many cellular processes [2].

The DUB/ubiquitin specific protease 17 (USP17) family of deubiquitinating enzymes were originally identified in mice as immediate early genes (DUB-1, DUB-1A, DUB-2) induced in response to a range of cytokines [3, 4]. Subsequently we demonstrated that expression of a human homologue, USP17/DUB-3/Dub3 (subsequently referred to as USP17), is induced in response to both cytokine and chemokine stimulation (IL-4, IL-6, IL-8, SDF1) [5, 6] and that USP17 expression is required for proper G1 to S cell cycle progression [7] and chemokine driven (IL-8, SDF1) cell motility [6]. In addition, others have shown that USP17 knockdown can impede tumour growth [8] and that USP17 expression is required to prevent the differentiation of embryonic stem cells (ESCs) [9]. Mechanistically, we have shown that USP17 regulates the activity of the protease Ras converting enzyme 1 (RCE1), which is required for the processing of ‘CaaX’ motif proteins such as H-Ras and N-Ras [10, 11] and most recently we have shown that this is mediated via the deubiquitination and re-localization of a novel isoform of RCE1 (RCE1 isoform 2) [12]. In addition, others have identified Cdc25A [8], SDS3 [13], RIG-I and Mda5 [14] as potential substrates for USP17.

Previously, we demonstrated that USP17 expression blunts EGFR signaling [11] indicating it has a role in the regulation of this pathway. In addition, we have shown that several cytokines and chemokines [5, 6] induce USP17 expression and we decided to examine if this was also the case for EGF. A study examining DUB-2A, a murine homologue of USP17, had indicated DUB-2A regulated the trafficking of colony-stimulating factor 3 receptor (CSF3R) to the lysosome and thus its down-regulation [15]. This prompted us to examine if USP17 had a similar impact on the trafficking of EGFR.

In this study we have now demonstrated that USP17 expression is induced in response to EGFR engagement and is required for EGFR endocytosis in the presence of low, but not high, concentrations of EGF. In addition, we have shown that USP17 depletion blocks the internalisation of transferrin and its receptor (TfR), an archetypal substrate for clathrin mediated endocytosis (CME). Furthermore, we demonstrate that in the absence of USP17 the plasma membrane recruitment of a number of components of the CME machinery is impaired. This data indicates that USP17 is required for EGFR and TfR endocytosis and that it plays a general role in CME.

## RESULTS

### USP17 is required for EGFR endocytosis

**FIGURE 1:**
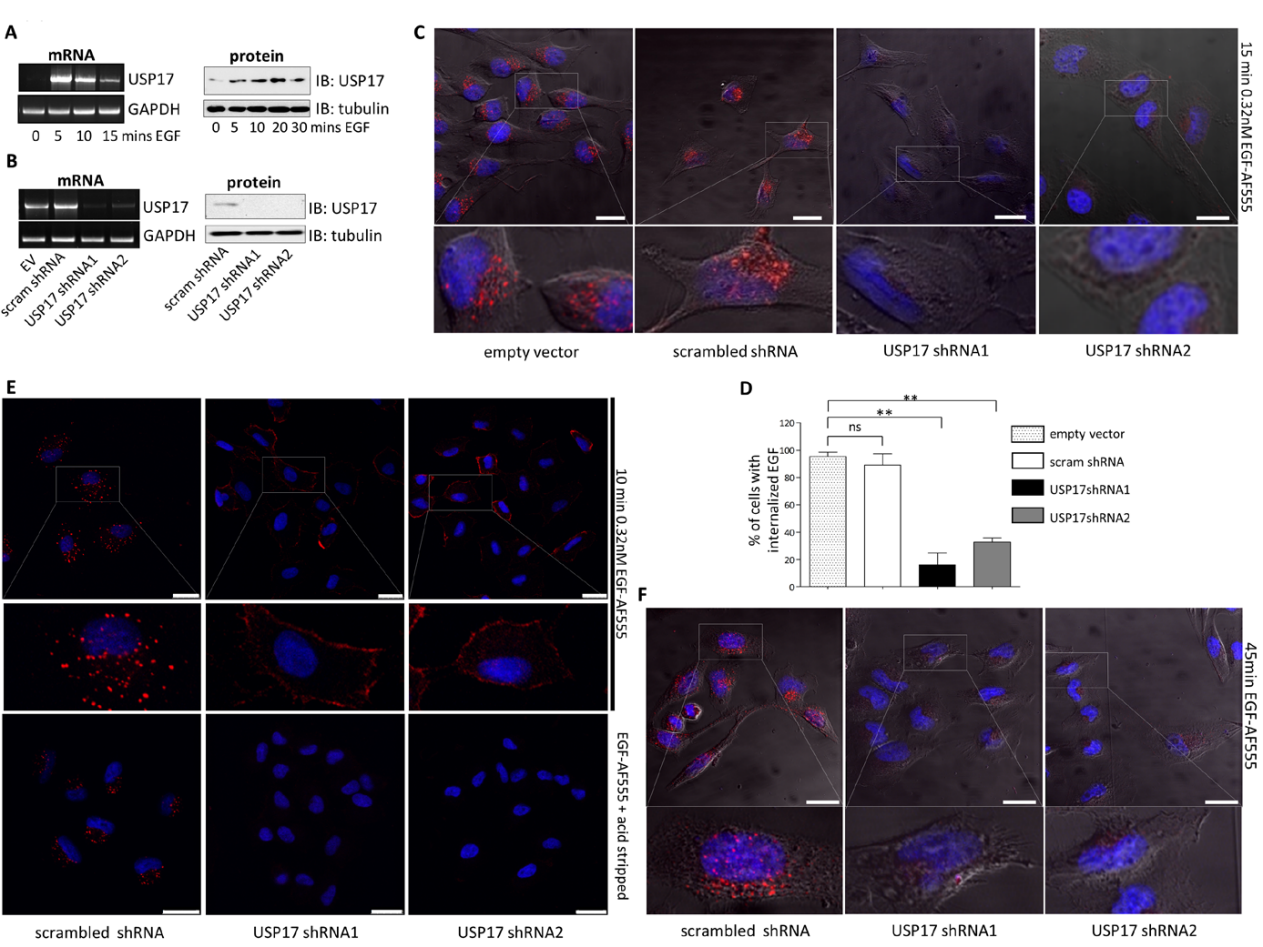
(a) HeLa cells were starved in serum free medium for 3 hrs prior to incubation with 0.32 nM EGF Alexa Fluor 555 and mRNA and protein samples were harvested at the time points indicated. USP17 and GAPDH (loading control) mRNA were then assessed by RT-PCR. USP17 protein levels were assessed by immunoblotting and tubulin was used as a loading control. (b) HeLa cells were transfected as indicated. 72 hrs post transfection mRNA and protein samples were harvested and USP17 mRNA and protein levels assessed as above. (c) HeLa cells were transfected with the constructs indicated. 72 hrs post transfection the cells were starved in serum free medium for 3 hrs prior to incubation with 0.32 nM EGF Alexa Fluor 555. After 15 min the cells were acid washed, fixed and the nuclei were stained with DAPI. EGF Alexa Fluor 555 (red) internalisation was then assessed in brightfield and fluorescent images taken using confocal microscopy. The bottom panels are enlarged images of the indicated area in the top panels. Scale bar = 20 μm. (d) At least 50 cells per condition were blindly scored for three separate experiments based on the presence of EGF Alexa Fluor 555 internalisation. (e) A549 cells were transfected as indicated and 72 hrs post transfection the cells were starved in serum free medium for 3hrs prior to incubation with 0.32nM EGF Alexa Fluor 555. After 10 mins the cells were either acid washed or washed with PBS. Subsequently they were fixed and the nuclei were stained with DAPI. EGF Alexa Fluor 555 (red) internalisation was then assessed in brightfield and fluorescent images taken using confocal microscopy. The middle panels are enlarged images of the indicated area in the upper panels. Scale bar = 25 μm. (f) HeLa cells were transfected as indicated and 72 hrs post transfection the cells were starved in serum free medium for 3 hrs prior to incubation with 0.32nM EGF Alexa Fluor 555. After 45 mins the cells were acid washed, fixed and the nuclei were stained with DAPI. EGF Alexa Fluor 555 (red) internalisation was then assessed in brightfield and fluorescent images taken using confocal microscopy. The lower panels are enlarged images of the indicated area in the upper panels. Scale bar = 10 μm. ** *p*<0.01

To determine if EGF induced USP17 expression we stimulated HeLa cells with EGF (0.32 nM) and observed a strong induction of both USP17 mRNA and protein (Fig. 1A) indicating that USP17 expression was indeed induced by EGF and this led us to further probe the role of USP17 in EGFR signaling.

To probe the role of USP17 in EGFR trafficking, we transfected HeLa cells with, either the previously validated USP17 specific shRNAs (shRNA1 and shRNA2) (Fig. 1B) [6, 7], a scrambled shRNA, or empty vector. We initially examined the internalisation of EGF Alexa Fluor 555 (0.32 nM) to see if any gross mis-localisation was evident. Control cells internalised the labeled EGF as expected and distinct vesicular structures containing EGF were evident (Figure 1C, panels 1-2). However, rather than altering the trafficking of EGF, USP17 depletion resulted in the failure of up to 80% of these cells to internalise EGF, something which was starkly illustrated by its absence when these cells were stripped of external EGF using an acid wash (Figs 1C panels 3-4, 1D). The same experiments were carried out using A549 cells and again, when USP17 was depleted, these cells failed to internalise any EGF (Fig. 1E, middle and right panels). However, if these cells were not subjected to an acid wash to strip the external EGF, the labeled EGF associated with their plasma membrane indicating that, although it was not internalised, it may still bind to EGFR (Fig. 1E, upper middle and upper right panels). To further confirm these observations were due to a loss of internalisation, and not a delay in this process, we extended the incubation period with EGF Alexa Fluor 555 out to 45 minutes and still observed no internalised EGF (Fig. 1F).

**FIGURE 2:**
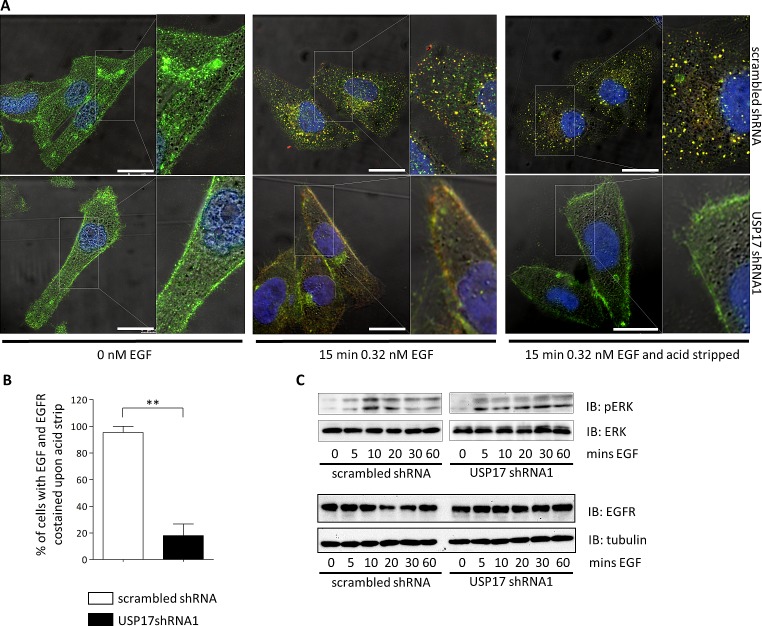
(a) HeLa cells were transfected as indicated. 72 hrs post transfection the cells were starved in serum free medium for 3 hrs prior to incubation with 0.32 nM EGF Alexa Fluor 555 where indicated. After 15 min the cells were acid washed, where indicated, fixed and the nuclei stained with DAPI. The cells were then stained using an anti-EGFR antibody and EGF Alexa Fluor 555 (red) and EGFR (green) internalisation was assessed in brightfield and fluorescent images taken using confocal microscopy. The right panels are enlarged images of the indicated area in the left panels. Scale bar = 10 μm. (b) At least 50 cells per condition were blindly scored for three separate experiments based on the presence of EGF Alexa Fluor 555 and EGFR co-staining post acid wash. (c) HeLa cells were transfected as indicated. 72 hrs post transfection the cells were starved in serum free medium for 3 hrs prior to incubation with 0.32 nM EGF. Whole cell lysates were harvested and levels of phosphorylated ERK1/2, ERK1/2, EGFR and tubulin were assessed by immuno-blotting using anti-pERK1/2, anti-ERK1/2, anti-EGFR and anti-tubulin antibodies. ** *p*<0.01

This indicated that USP17 was necessary for EGF entry. However, it could have also been explained by USP17 depletion resulting in the trafficking of EGFR away from the plasma membrane. Therefore, to investigate if USP17 was altering the location of EGFR we examined the localisation of EGF, along with EGFR, using an anti-EGFR antibody. In control HeLa cells we observed EGFR on the plasma membrane prior to stimulation, and upon EGF treatment, EGF and EGFR co-localised to internal vesicular structures which were more prominent after cells were stripped of external EGF using an acid wash (Figs 2A, top panels, 2B). In USP17 depleted cells we again observed prominent EGFR plasma membrane staining (Fig. 2A, bottom left panels). However, when EGF was added, both the EGF and EGFR remained at the plasma membrane and the EGF was lost upon acid wash demonstrating it had failed to enter the cell (Figs 2A, bottom middle and bottom right panels, 2B).

To further determine if the failure of EGFR to internalise was due to the loss of EGFR signaling, we examined ERK1/2 activation upon EGF treatment (Fig. 2C, top panels). ERK1/2 activation was observed regardless of the USP17 status indicating USP17 is not necessary for EGFR activation. We also examined EGFR protein levels and observed that the abundance of EGFR was not reduced by USP17 depletion (Fig. 2C, bottom panels). Taken together, this data clearly highlighted that USP17 is required for EGFR internalisation and indicated that the induction of USP17 by EGF allows EGFR internalisation.

### USP17 is not required for EGFR endocytosis in the presence of high EGF concentrations

**FIGURE 3:**
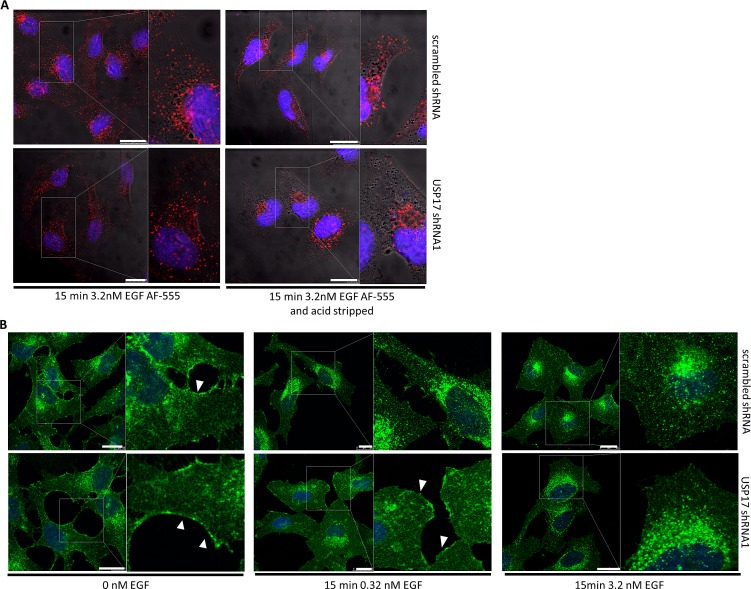
(a) HeLa cells were transfected as indicated. 72 hrs post transfection the cells were starved in serum free medium for 3 hrs prior to incubation with 3.2 nM EGF Alexa Fluor 555. After 15 min the cells were acid washed where indicated, fixed and the nuclei stained with DAPI. EGF Alexa Fluor 555 (red) internalisation was assessed in brightfield and fluorescent images taken using confocal microscopy. The right hand panels are enlarged images of the indicated area in the left hand panels. Scale bar = 10 μm. (b) HeLa cells were transfected with either scrambled shRNA or USP17 shRNA1 and 72 hrs post transfection the cells were starved in serum free medium for 3hrs prior to incubation with either 0 nM, 0.32 nM or 3.2 nM recombinant EGF. After 15mins the cells were washed, fixed and stained using an anti-EGFR antibody. EGFR (green) internalisation was assessed in fluorescent images taken using confocal microscopy. The right hand panels are enlarged images of the indicated area in the left hand panels. White arrows point out plasma membrane staining for EGFR. Scale bar = 20 μm.

EGFR internalisation proceeds mainly via CME [16], although clathrin independent pathways are also thought to be used if high EGF concentrations are present [17]. Therefore, to clarify which pathways are influenced by USP17 we examined the internalisation of EGFR under high EGF conditions. We transfected HeLa cells as before and examined the internalisation of EGF Alexa Fluor 555 using high EGF concentrations (3.2 nM) (Fig. 3A). In the presence of high EGF, USP17 depleted cells now showed the presence of internalised EGF in vesicular structures, although upon acid wash it was evident that these vesicles were localised in a peri-nuclear distribution, rather than throughout the cell as observed in the control cells (Fig. 3A, bottom panels). These observations were further confirmed using recombinant EGF (0.32 nM, 3.2 nM) and examining the localisation of EGFR using an anti-EGFR antibody (Fig. 3B). This again illustrated that EGFR endocytosis was blocked in cells lacking USP17 when low EGF concentrations were used (Fig. 3B, middle panels), but that high concentrations could overcome this block (Fig. 3B, right panels). These observations now suggested that USP17 was specifically required for the clathrin dependant internalisation of EGFR.

### Transferrin internalisation requires USP17

**FIGURE 4:**
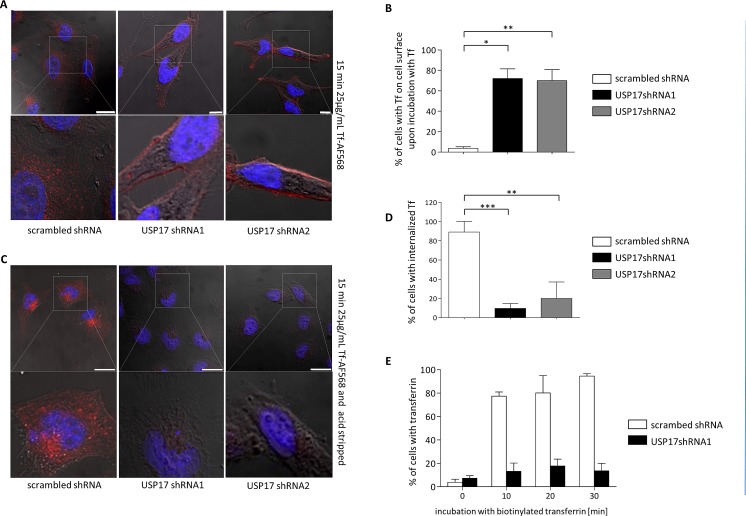
(a) HeLa cells were transfected as indicated. 72 hrs post transfection the cells were starved in serum free medium for 30 min prior to incubation with 25 μg/ml transferrin Alexa Fluor 568. After 15 min the cells were fixed and the nuclei stained with DAPI. The cells were then assessed for transferrin Alexa Fluor 568 (red) internalisation in brightfield and fluorescent images taken using confocal microscopy. The bottom panels are enlarged images of the indicated area in the top panels. Scale bar = 25 μm. (b) At least 50 cells per condition were blindly scored for three separate experiments based on the presence of transferrin Alexa Fluor 568 on the cell surface. (c) HeLa cells were transfected as indicated. 72 hrs post transfection the cells were starved in serum free medium for 30 min prior to incubation with 25 μg/ml transferrin Alexa Fluor 568. After 15 min the cells were acid washed, fixed and the nuclei stained with DAPI. The cells were then assessed for transferrin Alexa Fluor 568 (red) internalisation in brightfield and fluorescent images taken using confocal microscopy. The bottom panels are enlarged images of the indicated area in the top panels. Scale bar = 25 μm. (d) At least 50 cells per condition were blindly scored for three separate experiments based on the presence of transferrin Alexa Fluor 568 internalisation. (e) HeLa cells were transfected as indicated. 72 hrs post transfection the cells were starved in serum free medium for 30 min prior to incubation with 25 μg/ml biotin labeled transferrin (Sigma). After the indicated time points the cells were acid washed, fixed and stained using streptavidin-CY5 (BD Biosciences, USA). The cells were then assessed for transferrin internalisation using flow cytometry. * *p*<0.05, ** *p*<0.01, *** *p*<0.001.

Our data indicated that USP17 is required for clathrin mediated internalisation of EGFR, but it was unclear if USP17 played a role specific to EGFR, or if it might play a more general role in CME. Therefore, we now examined the impact of USP17 depletion upon the internalisation of transferrin, a proto-typical substrate for CME. HeLa cells were transfected as before and the internalisation of transferrin Alexa Fluor 568 was examined using confocal microscopy (Figs 4A-D) and FACS analysis (Fig. 4E). In both cases there was at least an 80% decrease in the number of cells showing the presence of internalised transferrin when USP17 was depleted indicating it was also required for transferrin internalisation (Figs 4B, D-E). Indeed, if the cells were not acid washed prior to visualisation, there was a prominent halo of transferrin observed around the cells depleted of USP17 indicating the transferrin was binding, but failing to internalise (Fig. 4A, middle and right panels).

**FIGURE 5:**
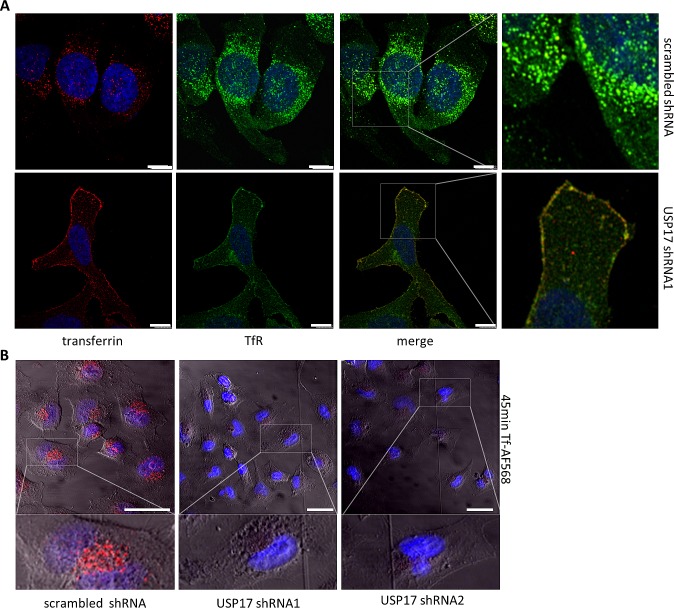
(a) HeLa cells were transfected as indicated. 72 hrs post transfection the cells were starved in serum free medium for 30 min prior to incubation with 25 μg/ml transferrin Alexa Fluor 568. After 15 min the cells were fixed and the nuclei stained with DAPI. The cells were then stained using an anti-TfR antibody and assessed for transferrin Alexa Fluor 568 (red) and TfR (green) internalisation in brightfield and fluorescent images taken using confocal microscopy. The right panels are enlarged images of the indicated area in the merged panels. Scale bar = 25 μm. (b) HeLa cells were transfected as indicated and 72 hrs post transfection the cells were starved in serum free medium for 3 hrs prior to incubation with 25 μg/ml transferrin Alexa Fluor 568. After 45 mins the cells were acid washed, fixed and the nuclei were stained with DAPI. Transferrin Alexa Fluor 568 (red) internalisation was then assessed in brightfield and fluorescent images taken using confocal microscopy. The lower panels are enlarged images of the indicated area in the upper panels. Scale bar = 10 μm.

We next examined the localisation of transferrin along with TfR using an anti-TfR antibody. In control cells we observed the majority of TfR on internal vesicular structures (Fig. 5A, top panels) and upon transferrin treatment we observed transferrin and TfR co-localised to these internal vesicular structures (Fig. 5A, top right panels). In contrast, USP17 depleted cells showed prominent TfR staining on the plasma membrane, which co-localized with transferrin and suggested TfR and transferrin both failed to internalise (Fig. 5A, bottom panels). Again, to confirm this was due to an internalisation block, and not a delay, we extended the incubation period with transferrin out to 45 minutes and still observed no internalisation (Fig. 5B). This now indicated that USP17 plays a more general role in CME as both EGFR (ligand triggered endocytosis) and TfR (constitutively recycles) require USP17 expression to internalise via CME.

### USP17 depletion impedes plasma membrane recruitment of CME machinery

Our work now indicated USP17 had a role in CME, but to further explore where USP17 acts, we examined the impact of USP17 depletion upon a number of the components required for CME.

**FIGURE 6:**
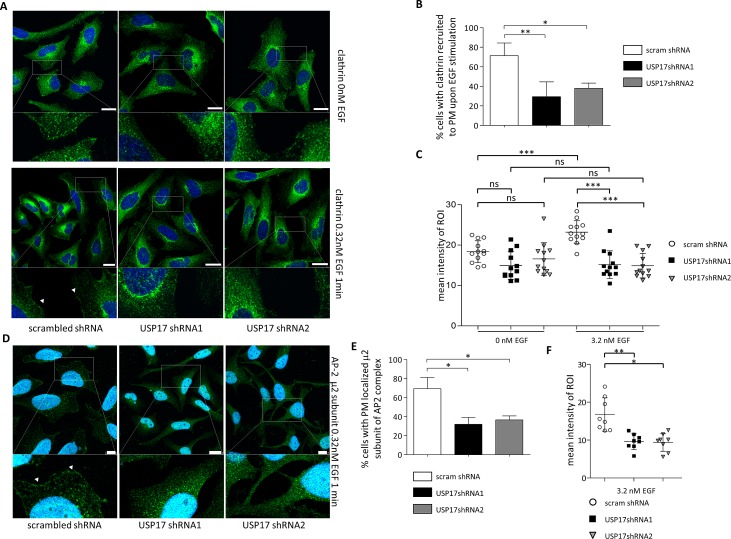
(a) HeLa cells were transfected as indicated. 72 hrs post transfection the cells were starved in serum free medium for 3 hrs prior to incubation with 0.32 nM recombinant EGF where indicated. After 1 min the cells were fixed and stained using an anti-clathrin antibody (green) and clathrin localisation was assessed in brightfield and fluorescent images taken using confocal microscopy. Bottom panels are enlarged images of the indicated area in the upper panels. Scale bar = 10 μm. (b) At least 50 cells per condition were blindly scored for two separate experiments based on the presence of clathrin at the plasma membrane. (c) Quantification of fluorescent signal intensity corresponding to endogenous clathrin within region of interest (ROI) defined as 15 pixel wide strip overlapping with boundary of the cell (detected in corresponding brightfield images). Intensities of the pixels within the ROI were quantified using Fiji software and expressed as mean intensity of ROI. ROIs of at least 10 representative cells for each condition were quantified and plotted. (d) HeLa cells were transfected as indicated. 72 hrs post transfection the cells were starved in serum free medium for 3 hrs prior to incubation with 0.32 nM recombinant EGF where indicated. After 1 min the cells were fixed and stained using an anti-μ2 subunit of AP-2 antibody (green) and AP-2 localisation was assessed in brightfield and fluorescent images. Bottom panels are enlarged images of the indicated area in the upper panels. Scale bar = 10 μm. (e) At least 50 cells per condition were blindly scored for two separate experiments based on the presence of AP-2 at the plasma membrane. (f) Quantification of fluorescent signal intensity corresponding to endogenous AP-2 within region of interest (ROI) defined as 15 pixel wide strip overlapping with boundary of the cell (detected in corresponding brightfield images). Intensities of the pixels within the ROI were quantified using Fiji software and expressed as mean intensity of ROI. ROIs of at least 8 representative cells for each condition were quantified and plotted * *p*<0.05, ** *p*<0.01, *** *p*<0.001.

Previous studies have indicated that EGF triggers clathrin recruitment to the plasma membrane within 1 minute [18] and we now transfected HeLa cells as before and examined clathrin localisation in the presence and absence of EGF using an anti-clathrin antibody (Figure 6A). In control cells, EGF treatment triggered the recruitment of clathrin spots to the periphery of the cell (Figs 6A, left panels, 6B, 6C). In cells lacking USP17 this recruitment was not evident (Figs 6A, middle and right panels, 6B, 6C) and treating these cells with EGF had no significant impact upon the amount of clathrin at the periphery (Fig. 6C).

We then examined the recruitment of AP-2, using an anti-μ2 subunit of AP-2 antibody, and observed that EGF again triggered the recruitment of AP-2 to the periphery of the cell in control cells (Figs 6D, 6E, 6F). However, this recruitment was again significantly reduced when USP17 was depleted (Figs 6D, 6E, 6F).

**FIGURE 7:**
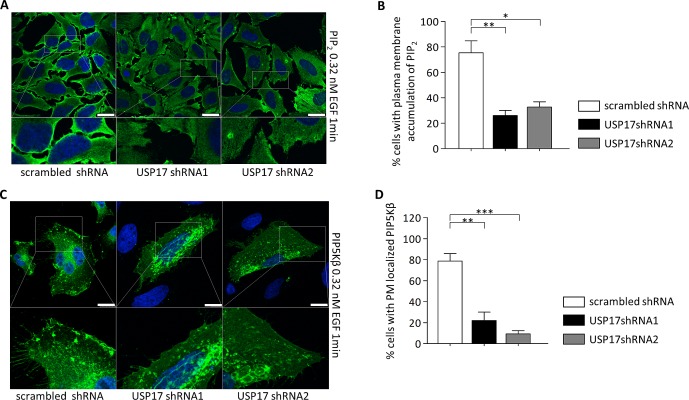
(a) HeLa cells were transfected as indicated. 72 hrs post transfection the cells were starved in serum free medium for 3 hrs prior to incubation with 0.32 nM recombinant EGF. After 1 min the cells were fixed and stained using an anti-PIP_2_ antibody (green) and PIP_2_ localisation was assessed in brightfield and fluorescent images taken using confocal microscopy. Bottom panels are enlarged images of the indicated area in the upper panels. Scale bar = 10 μm. (b) At least 50 cells per condition were blindly scored for two separate experiments based on the presence of PIP_2_ at the plasma membrane. (c) HeLa cells were transfected as indicated. 72 hrs post transfection the cells were starved in serum free medium for 3 hrs prior to incubation with 0.32 nM recombinant EGF. After 1 min the cells were fixed and stained using an anti-FLAG antibody (green) and PIP5K beta localisation was assessed in brightfield and fluorescent images. Bottom panels are enlarged images of the indicated area in the upper panels. Scale bar = 10 μm. (d) At least 50 cells per condition were blindly scored for two separate experiments based on the presence of PIP5K beta at the plasma membrane. * *p*<0.05, ** *p*<0.01, *** *p*<0.001.

Phosphoinositol-4, 5-phosphate (PIP_2_) is a lipid whose production at the plasma membrane allows the recruitment of many of the components of the CME machinery [19]. We also looked at the impact of USP17 knockdown on the localisation of PIP_2_, using an anti-PIP_2_ antibody, and we observed that USP17 loss resulted in a significant reduction in the PIP_2_ observed around the periphery of the cell upon EGF treatment (Figs 7A, middle and right panels, 7B). A number of enzymes are capable of producing PIP_2_ at the plasma membrane, however phosphoinositol-5-phosphate kinase beta (PIP5Kβ) has previously been shown to be necessary for EGFR and TfR endocytosis [20, 21]. Therefore, we looked at the localisation of a carboxy terminal FLAG-tagged PIP5Kβ and found that USP17 loss also resulted in a significant decrease in plasma membrane localisation of PIP5Kβ (Figs 7C, middle and right panels, 7D).

Collectively, this data now indicated that USP17 is required for CME as it allows the proper recruitment of the necessary machinery to the periphery of the cell.

## DISCUSSION

The data presented here demonstrates that USP17 expression is required for the internalisation of both EGF and EGFR in the presence of low level, but not high level EGF. This indicates that USP17 is required for the clathrin dependent internalisation of EGFR, something which is further confirmed by the demonstration that USP17 is also required for the internalisation of transferrin and TfR, archetypal substrates for clathrin mediated internalisation. In addition, this data also demonstrates that USP17 expression is necessary for the localisation of PIP5Kβ to the plasma membrane and therefore the production of PIP_2_ and the resulting recruitment of components of the CME machinery such as AP-2 and clathrin itself. In addition, when taken in conjunction with our previous observations that USP17 expression is induced in response to multiple stimuli [5, 6] our observations indicate that USP17 may play a fundamental role in regulating the internalisation of multiple receptor types and its induction may play a pivotal role in the regulation of the internalisation route selected.

This is the first report of a deubiquitinating enzyme being required for the internalisation of EGFR. However, other deubiquitinating enzymes such as AMSH and USP8 have previously been implicated in the regulation of EGFR endosomal trafficking after its internalisation [22]. In particular, EGFR has been shown to be ubiquitinated upon activation [23], prior to being internalised and trafficked to early endosomes from where it can either be recycled or sent to the lysosome for degradation [24]. AMSH can deubiquitinate EGFR and prompt recycling to the plasma membrane [25], whilst USP8 is required for the degradation of EGFR in the lysosome [26, 27, 28, 29]. AMSH and USP8 also play a similar role in regards to ERBB2, ERBB3, hepatocyte growth factor receptor (c-met), protease activated receptor 2, the delta-opioid receptor (DOR) and the chemokine receptor CXCR4 [29, 30, 31, 32, 33]. A number of other deubiquitinating enzymes have also been implicated in the regulation of endosomal trafficking [34], but to date this is the first report of such an enzyme being required for receptor internalisation.

We knew USP17 impacted upon EGFR signaling [11] and its induction by EGF led us to investigate this further. Another study indicating DUB-2A could impede the trafficking of CSF3R [15] prompted us in particular to examine its impact upon EGFR trafficking. However, rather than altering trafficking, we observed that USP17 loss blocked EGF and EGFR internalisation. Although unexpected, this fits with our previous observation that MEK1/2 and ERK1/2 activation was down-regulated upon USP17 over-expression [11] as the loss of EGFR internalisation results in enhanced Ras/MEK/ERK activation [35, 36].

The observation that higher concentrations of EGF could allow its internalisation in the absence of USP17 pointed to a role for USP17 specifically in clathrin mediated endocytosis as previous studies have indicated whilst EGFR is mainly internalised via a clathrin dependent mechanism [16, 37], a clathrin independent mechanism can be utilised in the presence of higher concentrations of EGF [38]. This was further supported by the observation that USP17 depletion blocked the recruitment of components of the CME machinery to the plasma membrane upon EGF stimulation and that transferrin internalisation was also lost upon USP17 depletion, as transferrin is regarded as a marker of this pathway. This impact on clathrin mediated endocytosis also potentially fits with our previous observations in regards to IL-8 and SDF1. In particular, the receptors for IL-8 (CXCR1/2) and SDF1 (CXCR4), like many other G-protein coupled receptors, internalise mainly via clathrin coated vesicles [39]. In addition, it has also been demonstrated that CXCR2 internalisation is required for CXCR2 driven chemotaxis [40, 41] with mutants of CXCR2 [41], or dynamin [40], which prevent internalisation blocking CXCR2 driven chemotaxis. Therefore, the previously observed loss of IL-8 and SDF1 driven chemotaxis in the absence of USP17 [6] would fit with the failure of the chemokine receptors to internalise upon engagement. In addition, the study examining DUB-2A [15] only examined DUB-2A over-expression and it is possible that the alterations in trafficking observed were due to DUB-2A over-expression triggering either earlier receptor internalisation, or altering the route utilised, and that DUB-2A is similarly required for receptor internalisation. Little is known in regards to the mechanisms responsible for the internalisation of the IL-4 and IL-6 receptors and it is difficult to elucidate the role of USP17 in these cases. However, it could be speculated that the induction of USP17 is again required to allow the internalisation and proper function of these receptors as both are known to internalise upon ligand engagement [42, 43].

The observation, that in the absence of USP17, the CME machinery doesn't localise properly to the plasma membrane, indicated that USP17 is required to allow CME as it allows the localisation of PIP5Kβ to the plasma membrane and thus the production of PIP_2_ and the recruitment of the CME machinery. Indeed, this would fit with our previous work on USP17 as it has been observed that Rac1 is required for PIP5Kβ localisation to the plasma membrane [44], Rac1, RhoA and Cdc42 activate PIP5K family members [45] and previously we have observed USP17 depletion blocks Rac1, RhoA and Cdc42 plasma membrane localisation [6, 7]. This would suggest that USP17 is required for Rac1, and thus PIP5Kβ localisation to the plasma membrane and thus CME. This connection to Rac1, a ‘CaaX’ motif protein, also suggests that the USP17 substrate responsible for this regulation may well be RCE1 isoform 2 [12] as it has previously been implicated in the processing of ‘CaaX’ motif protein [12]. Indeed, we have previously shown that over-expressing RCE1 isoform 2 can mimic the impact of USP17 depletion upon cell growth [12] indicating it is the USP17 substrate responsible for this effect. However, to confirm this is indeed the case, further studies will be required.

USP17 has also previously been shown to be necessary for proper cell cycle progression from G1 to S-phase and USP17 expression has been shown to be regulated in a cyclical fashion throughout the cell cycle [7]. It has been proposed that one, or a combination, of the previously identified USP17 substrates, RCE1 isoform 2 [10, 12], Cdc25A [8] or SDS3 [13] were responsible for its action upon the cell cycle. However, it has also been shown that clathrin mediated endocytosis is lost during mitosis [46], in particular during early mitosis [47] with clathrin potentially playing alternative roles during this period [48]. EGFR endocytosis has also been shown to be tightly regulated during the cell cycle and to be clathrin independent during M-phase [49]. Our observations could indicate regulation of USP17 could potentially contribute to the control of CME during the cell cycle and thus the regulation of the cell cycle itself.

The data presented here demonstrates that USP17 is required for CME of EGF and transferrin. It also indicates that USP17 plays a fundamental role in regulating the CME of multiple receptors and as such its induction may play a pivotal role in the regulation of receptor endocytosis and signalling. USP17 has also been suggested to represent a potential target for cancer therapy due to its over-expression in a number of tumour tissues [7], its association with recurrence and metastases in non-small cell lung cancer (NSCLC) [50], as well as its necessity for cell cycle progression [7], tumour growth [8] and cell migration [6]. However, its requirement for EGFR CME could make it an even more attractive target as EGFR signalling has been suggested to be more sustained when it is internalised and recycled via CME [51] and activating mutations of EGFR cause the receptor to be preferentially recycled back to the plasma membrane [52]. Therefore, inhibition of USP17 could potentially prevent EGFR CME, limiting its signalling capacity and driving it to the lysosome for destruction.

## MATERIALS AND METHODS

### Plasmids

pDQ-EV (His), pDQ-USP17 (His), and pDQ-USP17CS (His) were kind gifts from Dr. Derek Quinn (Queen's University, Belfast, UK). The pSUPER-USP17shRNA (USP17 shRNA1; target sequence 5′-GCAGGAAGATGCCCATGAA-3′), pRS-USP17shRNA (USP17 shRNA2; target sequence 5′-GATGATTTGGCTCCTGTGGCAAGACAGCT-3′) and pRS-scrambled shRNA were previously described [6].

### Cell Culture and DNA Transfections

HeLa cells (American Type Culture Collection (ATCC), Manassas, USA) were grown in DMEM supplemented with 10% FCS, 1% penicillin (10,000 U/ml) /streptomycin (10,000 μg/ml), and 1% L-glutamine (200 mM) (Life Technologies-Gibco, Paisley, UK). Cells lines were grown at 37^o^C in a 5% CO_2_ humidified incubator. Cells were transfected with FuGENE^TM^ 6 transfection reagent (Roche Diagnostics, Indianapolis, USA) according to manufacturer's instructions. Cells were seeded between 0.5 × 10^6^ and 1.0 × 10^6^ cells for protein experiments or 0.7-2.5 × 10^4^ on 4-well glass culture slides (BD Falcon, Bedford, USA) for microscopy experiments. The cells were transfected with 2μg of plasmid DNA for protein experiments and biological assays or 0.25 μg of plasmid DNA for confocal microscopy experiments. For those experiments with EGF stimulation, cells were rested for 3 hours in DMEM medium without serum. Cells were then stimulated with 0.32 nM-3.2 nM EGF Alexa Fluor 555 (Invitrogen-Molecular Probes, Eugene, USA) or recombinant human EGF (Invitrogen-Gibco, Maryland, USA) for the indicated times in the figures.

### EGF internalisation

Transfected cells were rested for 3hrs in DMEM medium without serum and stimulated with 0.32nM-3.2nM EGF Alexa Fluor 555 (Invitrogen-Molecular Probes, Eugene, USA) or recombinant EGF (Invitrogen-Gibco, Maryland, USA) for the indicated times in the figures. 0.32 nM and 3.2 nM EGF corresponded to the low (2 ng/mL) and high (20 ng/mL) EGF concentrations previously used [38]. Subsequently, to remove extracellular EGF cells were incubated with acid wash buffer (50 mM glycine, 150 mM NaCl, pH=3.0) for 5min at 37^o^C and washed with PBS (Life Technologies-Gibco, Paisley, UK) prior to fixing.

### Transferrin internalisation

Transfected cells were rested for 30 min in DMEM medium without serum. Cells were then incubated with 25 μg of transferrin Alexa Fluor 568 (Invitrogen-Gibco, Maryland, USA) for the indicated times in the figures. Extracellular Tf was removed as previously described for EGF internalization.

### Confocal Microscopy

HeLa and A549 cells were seeded at 0.7-2.5×10^4^ cells/1.7 cm^2^ well of glass culture slides (BD Falcon, Bedford, USA). Cells were transfected as previously described. The cells were fixed in 4% parafomaldehyde (Sigma-Aldrich, Steinheim, Germany), in PBS for 20 min. The cells were then permeabilized in 0.5% Triton X-100 in PBS for 5 min, washed in PBS and blocked in blocking solution (1% BSA, 10% donkey serum [both from Sigma, St. Louis, USA] in PBS) for 1 h at RT. Transfected proteins and cell organelles were stained with appropriate antibodies or counter stains according to manufacturer's protocol. Antibodies and co-stains were as follows: mouse anti-EGFR (GR01L, 1:1000, Merck-Calbiochem, Darmstadt, Germany), mouse anti-transferrin receptor (1:100, Invitrogen, Camarillo, USA), mouse anti-clathrin (1:300, Abcam, Cambridge, UK), mouse anti-AP-50 (1:200, BD Transduction Labs, USA), mouse anti-PIP_2_ (1:200, Enzo Life Sciences, New York, USA), mouse-anti-FLAG FITC (1:100, Sigma, St. Louis, USA), donkey anti-mouse Alexa Fluor 488 and donkey anti-rabbit Alexa Fluor 568 (both 1:200, Invitrogen-Molecular Probes, Eugene, USA). The slides were sealed with a coverslip and Prolong Gold antifade mounting media with DAPI (Life Technologies-Molecular Probes, Eugene, USA). Slides were viewed on a Leica SP5 Confocal Microscope. Fluorescent images were captured with a 63x lens zoomed 1-4x with a 1024×1024 frame and 400 Hz scanning speed. Images were analyzed using Leica LAS AF software. The images presented in the same figures were captured using standardized setting and exposure times.

### RNA extraction and reverse transcription-PCR

RNA was extracted using STAT-60 according to the manufacturer's instructions (Tel-Test Inc, Friendswood, USA). Reverse transcription-PCR (RT-PCR) was performed on 1 μg of total RNA using ImProm-II Reverse Transcription System (Promega, Madison, USA) as described previously [7]. The following primers were used: USP17, 5′-CAGTGAATTCGTGGGAATGGA GGACGACTCACTCTAC-3′ (forward) and 5′-AGTCATCGATCTGGCA CACAAGCATAGCCCTC-3′ (reverse). GAPDH 5′-ATGGCAAATTCCATGGCA-3′ (forward) 5′-TCTAGACGGCAGGTCAGG-3′ (reverse).

### Cell Lysis and Immunoblotting

Cells were lysed in the following buffer: 25 mM TrisHCl pH 7.6, 150 mM NaCl, 1% NP-40, 1% sodium deoxycholate, 0.1% SDS, supplemented with phenylmethylsulphonyl fluoride (1 mM), aprotinin (1.7 μg/ml) and leupeptin (10 μg/ml). Lysates were left on ice for 20 mins, centrifuged at 15,000 x g for 10 min at 4 ^o^C. Equal volumes of whole cell lysate were added to Laemlli buffer to a final concentration of 1X with 5% β-mercaptoethanol (Sigma, Germany). The samples were boiled for 5 min at 99^o^C for protein denaturation. The samples were analyzed by SDS-PAGE and Western blotting on PVDF membrane (Millipore, Waterford, UK). The membranes were then blocked in appropriate blocking agent, either 5% marvel or 3% BSA, in 0.1% Tween-20/PBS for 1 h. After blocking, the membranes were probed with the indicated antibodies for 1 hr at RT or overnight at 4 ^o^C. The following primary antibodies were used: rat anti-tubulin (1:10000, Abcam, Cambridge, UK), mouse anti-pERK1/2, mouse anti-ERK (both 1:1000, Cell Signalling, Danvers, USA), mouse anti-USP17 (Fusion Antibodies, Belfast, UK), mouse anti-EGFR (BD Biosciences, USA). The membrane was incubated with the appropriate secondary antibody: either goat anti-mouse HRP conjugate or goat anti-rabbit HRP conjugate (both diluted 1:10,000, BioRad, Hertfordshire, UK) or rabbit anti-rat HRP conjugate (1:40,000, Abcam, Cambridge,UK). Proteins were detected with a chemiluminescence protocol and were exposed using the ChemiDoc XRS+ imaging system (BioRad, Hercules, USA).
